# Comprehensive *in situ* co-detection of aneuploid circulating endothelial and tumor cells

**DOI:** 10.1038/s41598-017-10763-7

**Published:** 2017-08-29

**Authors:** Peter Ping Lin, Olivier Gires, Daisy Dandan Wang, Linda Li, Hongxia Wang

**Affiliations:** 1 Cytelligen, San Diego, CA USA; 2 Department of Otorhinolaryngology, Head and Neck Surgery, Grosshadern Medical Center, Ludwig-Maximilians-University of Munich, Marchioninistr. 15, 81377 Munich, Germany; 3 Clinical Cooperation Group ‘Personalized Radiotherapy in Head and Neck Cancer’, Helmholtz Zentrum München, German Research Center for Environmental Health GmbH, 85764 Neuherberg, Germany; 4 Department of Oncology, Shanghai General Hospital, Shanghai Jiao Tong University School of Medicine, Shanghai, China

## Abstract

Conventional circulating tumor cell (CTC) detection strategies rely on cell surface marker EpCAM and intracellular cytokeratins (CKs) for isolation and identification, respectively. Application of such methods is considerably limited by inherent heterogeneous and dynamic expression or absence of EpCAM and/or CKs in CTCs. Here, we report a novel strategy, integrating antigen-independent subtraction enrichment and immunostaining-FISH (SE-iFISH), to detect a variety of aneuploid circulating rare cells (CRCs), including CTCs and circulating tumor endothelial cells (CECs). Enriched CRCs, maintained at high viability and suitable for primary tumor cell culture, are comprehensively characterized by *in situ* co-examination of chromosome aneuploidy by FISH and immunostaining of multiple biomarkers displayed in diverse fluorescence channels. We described and quantified for the first time the existence of individual aneuploid CD31^+^ CECs and co-existence of “fusion clusters” of endothelial-epithelial aneuploid tumor cells among enriched non-hematopoietic CRCs. Hence, SE-iFISH is feasible for efficient co-detection and *in situ* phenotypic and karyotypic characterization as well as quantification of various CRCs, allowing for their classification into diverse subtypes upon biomarker expression and chromosome ploidy. Enhanced SE-iFISH technology, assisted by the Metafer-iFISH automated CRC imaging system, provides a platform for the analysis of potential contributions of each subtype of CRCs to distinct clinical outcome.

## Introduction

Non-hematopoietic circulating rare cells (CRCs) mainly consist of circulating tumor cells (CTCs) and circulating endothelial cells (CECs). Clinical relevance of CECs in tumor angiogenesis^1^ and CTCs in tumor metastasis^2, 3^ have been substantially discussed elsewhere.

Aneuploidy, leading to genomic instability^4^, is the most common characteristic of malignant cells^5, 6^. In addition to aneuploid neoplastic cells in the tumor mass, aneuploid CTCs in carcinoma patient blood were previously reported by us and others^7, 8^. Though aneuploid CD31^+^ tumor endothelial cells were detected in tumor tissue^9^, actual occurrence of aneuploid CD31^+^ CECs in circulation remains unknown.

Most of the current CRC detection technologies rely on cell surface molecules for isolation, and antibody staining of cellular proteins for identification^10^. However, constant or dynamic heterogeneity^11, 12^, which might result in complete absence of the anchor protein(s) targeted for detection, significantly interferes with isolation or identification of CRCs, respectively. Particularly, with respect to CTC detection, most of the conventional methodologies are biased towards detection of the only EpCAM and cytokeratin (CK) double-positive CTCs^10^. However, inherently heterogeneous and dynamic expression of EpCAM^11^, revealed by both microscopic immunofluorescence imaging^13^ and flow cytometry analyses^14^, as well as down-regulation of CK^15^ during epithelial-to-mesenchymal transition (EMT)^12^, inevitably lead to non-negligible false negative detection of such “uncapturable and invisible” CTCs. It is therefore imperative to develop an alternative strategy, aside from CK and EpCAM or relative proteins alone, for effective detection of the full spectrum of heterogeneous CECs and CTCs.

In the present report, we extended our previous prototyping study^13, 14^ to significantly improve the unique strength of SE-iFISH, which was developed to combine *in situ* phenotypic identification of tumor biomarker and karyotypic characterization of chromosome ploidy in CRCs enriched from patient biofluids (blood, bone marrow, ascites, malignant pleural effusion, and cerebrospinal fluids, etc.) by means of subtraction enrichment, independently of cell size, cluster or surface anchor protein expression. Comparing to the initial strategy restricted to only a single biomarker, the current significantly improved expeditious SE-iFISH, not only provides a comprehensive solution for selection and culture of primary tumor cells enriched and maintained at high viability, but also maximizes flexibilities of the existing iFISH to investigate whatever chromosome or multiple biomarkers in aneuploid CTCs at a time, despite their subcellular localization. Moreover, the isolated intact single iFISH CTC, not subjected to laser incision, is suitable for downstream whole genome amplification (WGA) and next generation sequencing (NGS) analysis.

Besides localized in tumor tissue^9, 16^, non-hematopoietic aneuploid CD31^+^ endothelial cells were detected for the first time in peripheral blood in this report, demonstrating the existence of aneuploid CD31^+^ tumor CECs. Heterogeneous expression of CD31 molecules on CECs and some CTCs, as well as circulating tumor microemboli (CTM) reported in this study, suggests that aneuploid CD31^+^ cells under pathologic circumstances may have diverse characteristics comparing to the conventional categories of CD31^+^ cells. Extensive additional investigation, regarding individual CTCs, tumor CECs, and co-existence of “epi-endo fusion-clusters” of aneuploid CTCs and CECs, are acquired to shed light on a potential functional interplay of diverse subtypes of aneuploid CRCs in tumor angiogenesis and metastasis.

## Results and Discussion

### Subtraction Enrichment (SE) and iFISH

EpCAM-dependent capture, tumor cell size-based filtration^17^ and immunostaining of intracellular CKs currently constitute the most common strategies for isolation and identification of CTCs, respectively^10, 13^. However, it has been concerned that besides existence of significant amount of small size CTCs^18^, such as EMT CTCs^19^, which may escape from cell filtration detection^20^, expression of EpCAM on CTCs and throughout tumor progression in general, is heterogeneous and dynamic even among individual CTCs within the same patient. Heterogeneity can result from either differing localizations of EpCAM within cells^13, 14^, or a loss of CK and EpCAM during EMT^11, 21^. In view of the reality that both EpCAM positive and negative CTCs have a potential to metastasize^22, 23^, and EMT impacts on response to standard therapy^21, 22^, hence, enrichment of systemic cancer cells independently of EpCAM or other markers, which might be prone to regulation, is of paramount importance to improve the functional analysis of these clinically relevant malignant cells. In addition, because intracellular signaling pathways of neoplastic cells could be activated by crosslinking of cell surface molecules (such as EpCAM) following antibody binding^24–26^, it is not surprised that subsequent analyses of intracellular signaling events in CTCs isolated by anti-EpCAM strategies might result in post-collection artifacts due to anti-EpCAM perturbing. It is, therefore, necessary to develop novel EpCAM/CK-independent strategies to effectively detect CTCs, regardless of type and stage of cancer.

**Figure 1:**
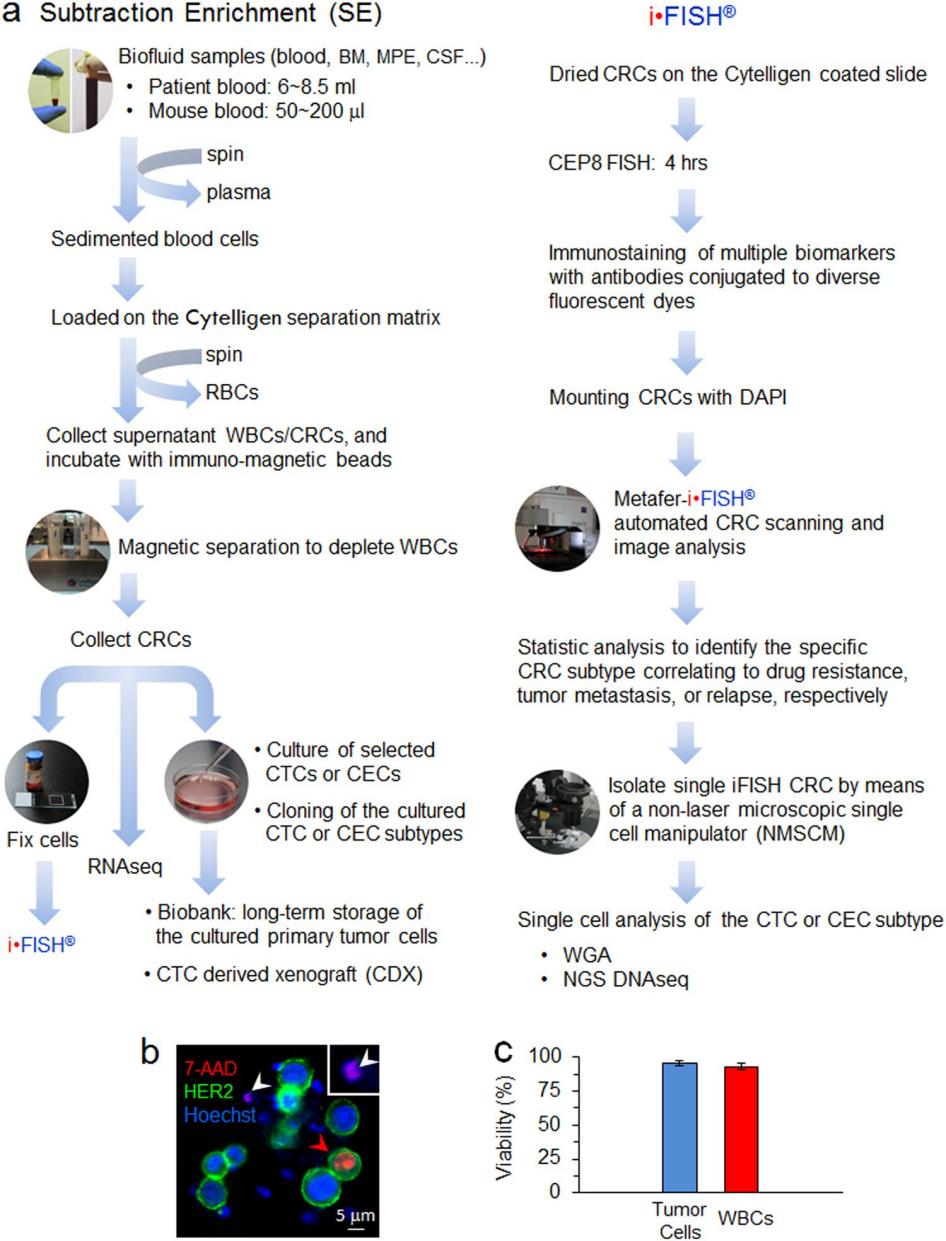
Schematic diagram of SE-iFISH and viability examination of the enriched tumor cells. (**a**) Illustration of schematic experimental flow and application of SE-iFISH. Diverse subtypes of CRCs in tumor animal models or cancer patients’ biofluid samples, including blood, bone marrow (BM), ascites, malignant pleural effusion (MPE), and cerebrospinal fluid (CSF), etc., are effectively and efficiently enriched, identified and characterized. Obtained viable CRCs including CTCs are available for primary tumor cell culture and a series of indicated downstream analyses. (**b**) Viability examination of tumor cells and WBCs following subtraction enrichment and immunostaining processing. Necrotic breast cancer cell (7-AAD^+^/HER2^+^/Hoechst^+^) (red arrow), and WBC (7-AAD^+^/HER2^−^/Hoechst^+^) (white arrow) are depicted. The same enlarged necrotic WBC is shown in the top right corner box. Healthy cells are 7-AAD negative. (**c**) Average of viability of the enriched tumor cells (blue) and WBCs (red) is 95.3 ± 2% and 93 ± 2.1% (mean ± SD), respectively. Results represent average of values obtained in 3 separate experiments performed in triplicate. Cytelligen, the copyright holder of the registered iFISH trademark, ensures Nature Publishing Group (NPG) to publish iFISH logo image in all formats under an Open Access License.

In the present study, we extended our previous efforts to significantly improve the prototyping subtraction enrichment and iFISH^13, 14^, respectively. Figure 1a schematically depicts the increased CRC detection efficiency and expanded applicable utilities of the evolved SE-iFISH.

Taking advantage of our hands-on experience to separate and isolate subcellular compartments as previously performed with discontinuous sucrose gradient centrifugation^27, 28^, a novel non-hematopoietic cell separation matrix of specific density was developed to effectively and rapidly deplete RBCs from nucleated WBCs and CRCs via centrifugation. The newly developed matrix is non-toxic to cells and does not apply hypotonic damage to the target cells. Moreover, in contrast to CD45-dependent negative enrichment, application of unique immuno-magnetic beads conjugated to antibodies specific for multiple WBC surface antigens makes SE feasible to efficiently deplete most of WBCs (approx. 4~5 logs).

SE-captured CTCs are viable and suitable for subsequent primary CTC culture. Shown in Fig. 1b, following enrichment and immunostaining processing, very few necrotic cells as determined with 7-AAD were revealed. Both enriched tumor cells and residual WBCs showed high viability of 95.3 ± 2% and 93 ± 2.1% (mean ± SD, Fig. 1c), respectively, indicating that SE had no significant detrimental impact on CTC viability. In addition to enumerating CTC, examination of CTC viability by means of 7-AAD, which stains nuclei of necrotic cells, may provide an additional choice to evaluate *in vivo* or *in vitro* (chemo)therapeutic efficacy on the enriched or cultured primary tumor cells. Our recent joint clinical study (UCSD Moores Cancer Center, San Diego, CA, USA) demonstrated that repeated freezing-and-thawing didn’t affect viability of the cultured and stored primary tumor cells enriched by SE from ascites of cancer patients. Moreover, demonstrated in Fig. 1a, application and significance of CTC derived xenograft/explant (CDX)^29, 30^ or cell line^30^ were published and discussed by others. Future cloning of cultured primary tumor cells classified into diverse subtypes relevant to the specific clinical outcome by iFISH, will provide additional meaningful approaches for cancer study.

Comparison of SE *vs* a classical capturing strategy performed with anti-EpCAM antibody immobilized on solid phase (*CellSearch*) showed that, despite heterogeneous and variable EpCAM expression, SE stably maintained a higher recovery efficiency on spiked cancer cells, including breast ^EpCAM+++^, bladder ^EpCAM+^, and melanoma ^EpCAM−^, as well as higher CTC detection positivity on gastric cancer patients (90.5% of SE *vs* 54.8% of anti-EpCAM by *CellSearch*)^7, 14^. Besides, our previous preliminary quantification study performed on chemotherapeutic lung cancer patients indicated that, quantified CTCs correlated well with patients’ clinical responses classified by CT scanning^31^. Additional study revealed that enumerated CTCs enriched from lung cancer patients were relative to the quantity of Cyfra 21–1 derived from CK19 in patients’ blood (cut-off value: 3.3 ng/ml)^32^.

The established iFISH strategy makes it feasible to perform *in situ* phenotyping and karyotyping of CTCs. In addition to providing higher sensitivity and specificity for CTC detection, quantification of chromosome ploidy performed by iFISH enables classifying CTCs into diverse subtypes upon tumor biomarkers expression and chromosome ploidy. Our recent study suggested that the specific subtype of CTCs possessing triploid chromosome 8 in gastric cancer patients, might have intrinsic resistance to the chemotherapeutic agent cisplatin, whereas tetra- and pentaploid subtype of CTCs seemed to have acquired resistance to cisplatin^7^. Similar results regarding specific subtype of CTCs resistant to cisplatin were obtained from our joint study performed on the gastric neuroendocrine cancer patient derived metastatic xenograft model (mPDX)^33^. In addition, correlation of diverse chromosome aneuploidies of CTCs with poor progression-free survival (PFS) and overall survival (OS) in gastric cancer patients has been recently published^34^. Identification of the specific CTC subset correlating with distinct clinical endpoint by iFISH, will help guide more meaningful studies (such as NGS analysis) performed on the targeted single CTC enriched from different types of cancer patient or tumor animal models. Preliminary EGFR mutation analysis of the targeted individual lung cancer CTCs was reported by us^35^. By means of a non-laser microscopic single cell manipulator (NMSCM, Cytelligen) to isolate single iFISH CTC, NGS analyses of the subtyped individual CTCs with distinct clinical relevance are currently performed by us on a large cohort of carcinoma patients.

Metafer-iFISH^®^, an automated CRC scanning and image analysis system, was recently co-developed by Carl Zeiss (Oberkochen, Germany), MetaSystems (Altlussheim, Germany) and Cytelligen (San Diego, CA, USA) in this report. High through-put X-Y-Z 3-dimensional slide scanning, image acquisition, processing, classification of CRC subtypes, and statistical analyses of each subcategory of the target cells could be fully automatically performed by Metafer-iFISH upon cell size, cell cluster, quantified chromosome ploidy, and immunofluorescence staining intensity of multi-tumor biomarkers expression displayed in diverse fluorescence channels, avoiding interference from human error or bias. Moreover, permanently saved digital coordinates of the precise location for each identified CRCs, enables scientists to isolate intact single target cell with high efficiency using a non-laser microscopic single cell manipulator (NMSCM). Metafer-iFISH^®^, constantly improved and validated on vast amount of clinical samples, indeed accelerates SE-iFISH to perform more sophisticated detection and characterization of CRCs in a large cohort of patients.

### Comprehensive Detection, *in situ* Phenotypic and Karyotypic Characterization of Tumor Cells Enriched from Blood

Our previous prototyping iFISH was restricted to simultaneously co-examine only one biomarker and cancer cell chromosome ploidy at a time^14^. Such restriction significantly limited scientists to comprehensively investigate how multiple biomarkers interplay in/on highly heterogeneous CRCs and how they further correlate with clinical outcomes in carcinoma patients. In the current study, we took advantage of our previously established immunofluorescence staining strategies suitable for cytosolic or membrane associated proteins with various localizations, such as plasma membrane, subcellular organelle membrane, cytosol or in nucleus^27, 36^, to successfully develop a comprehensive multi-marker-iFISH strategy. Great efforts were made in this study to optimize antibody conjugation strategies for diverse fluorochromes with adequate emission wavelengths, as well as FISH processing adapted immunofluorescence staining procedures suitable for varieties of cellular antigenic epitopes, to allow detection of combined cellular biomarkers such as HER2, Vimentin, PD-L1, PSA, CD133, CD44v6, GFAP, CA19-9, CA125, AFP, methothelin, DR4, CKs, and EpCAM, etc. Such improvement, expanding from previous four-color mono-marker iFISH to the current six-color tri-marker iFISH, including two invisible far infrared tracers, enables current iFISH to examine whatever tumor biomarker in karyotyped CRCs, despite differential subcellular localization.

**Figure 2:**
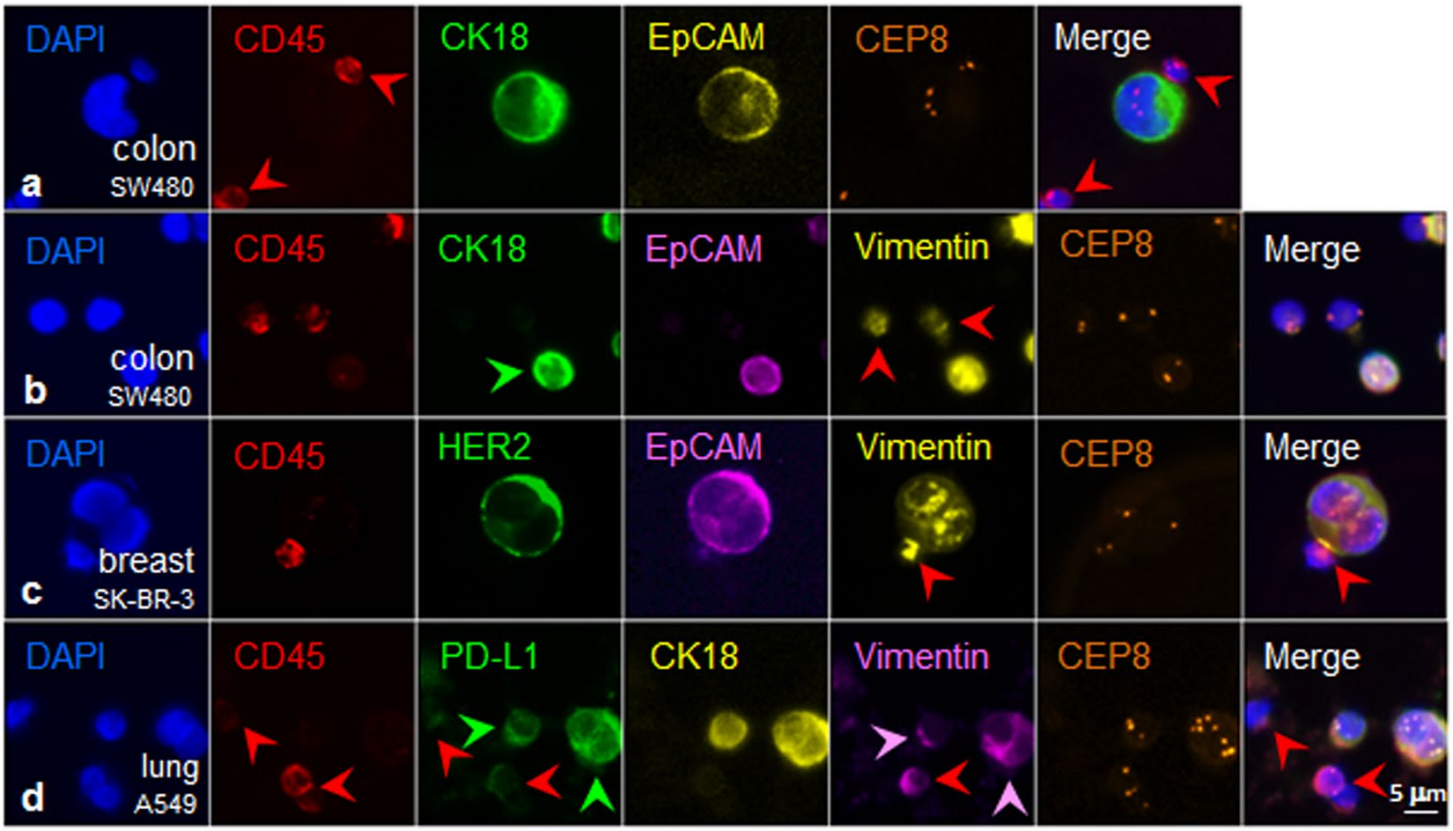
Multiple tumor biomarkers-iFISH. (**a**) (CK/EpCAM)-iFISH performed on enriched triploid colon cancer cell shows CD45^−^/CK18^+^/EpCAM^+^. WBCs (red arrows) are CD45^+^/CK18^−^/EpCAM^−^. (**b**) Tri-marker-iFISH indicates that the diploid tumor cell (green arrow) is CD45^−^/CK18^+^/EpCAM^+^/Vimentin^+^. Two of diploid WBCs (red arrows) are CD45^+^/Vimentin^+^. (**c**) (HER2/EpCAM/Vimentin)-iFISH reveals diploid breast cancer cell CD45^−^/HER2^+^/EpCAM^+^/Vimentin^+^. The WBC attached to the tumor cell is CD45^+^/Vimentin^+^ (red arrow). (**d**) Two of aneuploid lung cancer cells are CD45^−^/PD-L1^+^/CK18^+^/Vimentin^+^ (pink arrows). Depicted WBCs (red arrows) are CD45^+^/PD-L1^+^/CK18^−^, and one of WBCs is strong vimentin^+^ (red arrow).

As a proof-of-concept of the maximized SE-iFISH capability to co-detect expression of CK and EpCAM, which are of dual marker properties of both biomarker and epithelial marker in the same aneuploid tumor cell, colon cancer cells SW480 were spiked into human blood, followed by subtraction enrichment and dual biomarker (CK/EpCAM)-iFISH. Non-hematopoietic tumor cells displayed CD45^−^/CK18^+^/EpCAM^+^ along with trisomy of chromosome 8, as obtained with the Metafer-iFISH^®^ Automated CRC Image Scanning and Analyzing System (Fig. 2a).

Vimentin, another dual marker in neoplastic cells, is a protein possessing unique biofunctional properties of being both a crucial tumor relevant intracellular biomarker and a typical mesenchymal marker for critical EMT in CTCs^21, 37, 38^. To further examine EMT status of tumor cells, an expanded tri-marker (CK/EpCAM/Vimentin)-iFISH was developed and performed on SW480 cells enriched from blood. Vimentin is an intermediate filament protein expressed in cells of mesodermal origin, and in CTCs which have undergone EMT. Vimentin is frequently used to define the EMT status of carcinoma cells. Shown in Fig. 2b, results indicated that the SW480 tumor cell line comprised CD45^−^/CK18^+^/EpCAM^+^/Vimentin^+^ cells. Another set of tri-marker (HER2/EpCAM/Vimentin)-iFISH performed on the enriched SK-BR-3 breast cancer cells demonstrated that tumor cells display a CD45^−^/HER2^+^/EpCAM^+^/Vimentin^+^ phenotype (Fig. 2c). Imaging of tri-marker (PD-L1/CK18/Vimentin)-iFISH on A549 lung cancer cells exemplified two aneuploid CD45^−^/PD-L1^+^/CK18^+^/Vimentin^+^ cancer cells (Fig. 2d). Hence, using variations of biomarkers, *in situ* immune-phenotyping and karyotyping of rare CRCs in the blood is feasible.

### Characterization of Aneuploid CD31^+^ CECs and Comprehensive *in situ* Co-detection of Aneuploid CECs and CTCs

In view of the significant relevance of CECs^1, 9, 39, 40^ and CTCs^22, 29^ to cancer prognosis, tumor angiogenesis and metastasis formation, we utilized SE-iFISH to characterize aneuploid CD31^+^ CECs, and comprehensively co-detect CTCs and CECs in patient blood.

During endothelial lineage differentiation, early circulating endothelial CD31^+^/CD34^+^/CD133^+^ progenitors (CEPs) phenotypically down-regulate CD133 expression and increase CD31 expression to differentiate into matured CD31^+^/CD34^+^/CD133^−^ CEPs^41^, and then to matured CD31^+^/CD146^+^ conventional CECs^1, 41^. Besides, existence of another subset of CD31^+^/CD105^+^ cells was also reported^42^.

With respect to cytogenetics of CECs, in contrast to normal diploid endothelial cells, “tumor endothelial cells” identified in solid tumor mass are cytogenetically abnormal, showing aneuploidy of CD31^+^ cells^9^. Though extensive studies of CECs were published, none of those has addressed cytogenetic abnormality of tumor CECs in circulation of carcinoma patients.

**Figure 3:**
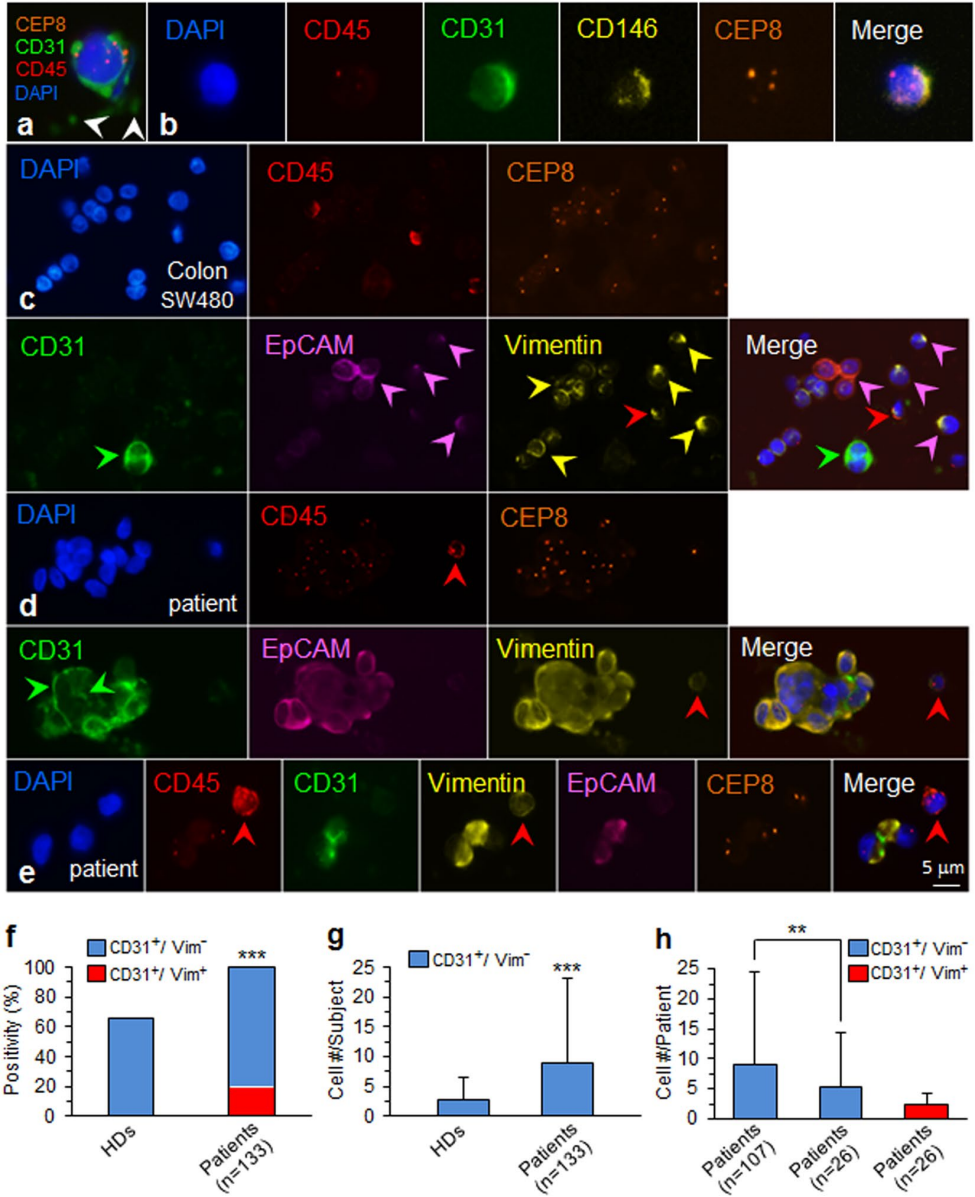
Characterization of aneuploid CECs and *in situ* co-detection of aneuploid CTCs and CECs. (**a**) An aneuploid CD45^−^/CD31^+^ CEC in a breast cancer patient is depicted. Surrounding platelets are indicated by white arrows. (**b**) The enriched triploid circulating CD31^+^ cell is CD45^−^/CD146^+^. (**c**) Aneuploid non-hematopoietic colon cancer cells (CD45^−^) spiked in a healthy donor blood show heterogeneous expression of EpCAM^+^ (pink arrows) and vimentin^+^ (yellow arrows). One of CD45^+^ WBCs is vimentin^+^ (red arrow). A cluster of 2 endogenous CECs (green arrow) is CD45^−^/CD31^+^/EpCAM^−^/Vimentin^−^. (**d**) A CTM detected in a patient with urologic malignancies shows heterogeneous expression of CD31 both along the edge of CTM and on the clustered tumor cells (green arrows), which are also EpCAM^+^/Vimentin^+^. The WBC (red arrow) is CD45^+^/Vimentin^+^/CD31^−^. (**e**) Two tumor cells (diploid and triploid of each), detected in the same cancer patient, show CD45^−^/CD31^+^/EpCAM^+^/Vimentin^+^. The WBC (red arrow) is CD45^+^/Vimentin^+^/CD31^−^. (**f**) Positivity analysis: 31 out of 47 healthy donors (HDs) (66%), and all of 133 carcinoma patients (100%) have aneuploid CD31^+^ CECs detected. The difference is statistically significant (***p < 0.001, chi-square test). Among 133 CEC positive cancer patients, 19.5% of subjects (26/133) have the mixed phenotypes of CD31^+^/Vim^+^ double positive and CD31^+^/Vim^−^ single positive aneuploid CECs (red), whereas remaining 80.5% (107/133) only have the CD31^+^/Vim^−^ single-positive phenotype CECs (blue). None of CD31^+^/Vim^+^ double positive CECs was detected in HDs. (**g**) Cell number analysis: the average CD31^+^/Vim^−^ aneuploid CEC number/subject is 2.8 ± 3.6 cells (Mean ± SD)/HD (n = 47), and 8.8 ± 14.3 cells/patient (n = 133). The difference is statistically significant (***p < 0.001, Mann-Whitney U test). (**h**) 107 patients with CD31^+^/Vim^−^ single-positive phenotype CECs have an average cell number of 9.1 ± 15.3 CECs/patient (blue). Remaining 26 patients who have mixed phenotypes, show average cell number of 5.4 ± 8.9 single-positive CECs (CD31^+^/Vim^−^)/patient, and 2.3 ± 1.8 double-positive CECs (CD31^+^/Vim^+^)/patient (red), respectively. The difference in cell number of the same single-positive phenotype CECs (CD31^+^/Vim^−^)/patient, between two different populations of patients (107 *vs* 26), was statistically significant (**p < 0.01, Mann-Whitney U test). No statistical difference in cell number/patient is observed between two diverse phenotype of CECs (CD31^+^/Vim^−^ *vs* CD31^+^/Vim^+^) in the same population of 26 patients.

We performed iFISH to phenotypically and karyotypically examine blood samples of cancer patients and healthy blood donors (HDs). CD31^+^ CECs with aneuploid chromosome 8 (Fig. 3a) were detected in both HDs and cancer patients, indicating existence of aneuploid CECs for the first time. Further characterization illustrated that less than 5% of the identified aneuploid CD31^+^ CECs express CD146 (Fig. 3b) or CD34 in both cancer patients (n = 6) and healthy blood donors (n = 8). Additional phenotypic characterization indicated that the detected CD31^+^ CECs were neither CD133^+^ nor CD105^+^ (data not shown). Obtained results suggest that non-hematopoietic CD31^+^ cells detected in this study were heterogeneous, which may include few CEPs (CD31^+^/CD34^+^) and mature CECs (CD31^+^/CD146^+^), as well as the so far non-reported novel CD31^+^/CD34^−^/CD133^−^/CD105^−^/CD146^−^ subtype. This novel subcategory of CECs contributes the most (95%) to the overall aneuploid CD31^+^ CECs, detected in both cancer patients and healthy blood donors in this preliminary characterization study performed on a small cohort of subjects.

To investigate if SE-iFISH is able to karyotypically and phenotypically co-detect aneuploid CECs and CTCs with different EMT status, as a proof-of-concept, SW480 colon cancer cells were spiked into healthy donor blood, followed by subtraction enrichment and (CD31/EpCAM/Vimentin)-iFISH. Shown in Fig. 3c, a cluster of 2 endogenous CD31^+^ CECs (green arrow) was co-detected with non-hematopoietic tumor cells (CD45^−^/CD31^−^/EpCAM^+^/Vimentin^+^) (pink arrows).

In view of the obvious biological and clinical significance of vimentin in cells, clinical validation of co-detecting vimentin, EpCAM and CD31 on CECs as well as CTCs was performed on a relative large cohort of 133 cancer patients. Besides individual aneuploid CTCs^7, 14^ and CD31^+^ CECs (Fig. 3a) detected, circulating tumor microemboli (CTM) was detected in this study. Similar to the reported tumor endothelium-derived circulating tumor endothelial cell clusters in colorectal cancer patients^43^, which could co-express both endothelial and mesenchymal biomarkers, a heteroploid CTC cluster (CTM) (Fig. 3d), enriched and identified in a patient with urologic neoplasm, showed a unique phenotype of CD45^−^/CD31^+^/EpCAM^+^/Vimentin^+^, with distinct localization of CD31, both along the edge of the cell cluster, and heterogeneously distributed on the clustered cancer cells (green arrows). Examination of an additional CTM (Fig. 3e), enriched from the same patient and composed of two cells, demonstrated that both tumor cells were CD45^−^/CD31^+^/EpCAM^+^/Vimentin^+^, indicating existence of non-reported “aneuploid endothelial-epithelial fusion cluster” in cancer patients.

To better understand how aneuploid CD31^+^ CECs distributed in both healthy blood donors (HDs) and cancer patients, and how endothelial marker CD31 as well as mesenchymal marker vimentin (Vim) co-localized on cells in cancer patients, statistical analyses were performed on both, the same phenotype of CD31^+^/Vim^−^ aneuploid CECs in different population of subjects, and the diverse phenotypes of CD31^+^/Vim^−^ *vs* CD31^+^/Vim^+^ aneuploid CECs in the same population of patients. Data quantification is depicted in Fig. 3f. Among 47 HDs, 66% of subjects (31/47) had CD31^+^ aneuploid CECs detected, and 100% of 133 cancer patients contained CD31^+^ aneuploid CECs in the blood (133/133, blue). Differences in CD31^+^ aneuploid CECs quantities between HDs and cancer patients were statistically significant (*p* < 0.001). Additional analysis indicated that among those 133 patients, there were two diverse populations of subjects identified, showing different phenotypes of aneuploid CECs, including 107 patients only with CD31^+^/Vim^−^ single positive phenotype aneuploid CECs (107/133 = 80.5%, blue), and 26 patients with mixed phenotypes of CD31^+^/Vim^+^ double positive and CD31^+^/Vim^−^ single positive aneuploid CECs (26/133 = 19.5%, red). The CD31^+^/Vim^+^ double positive CECs were not detected in HDs.

Quantification of CECs per subject revealed an average 2.8 ± 3.6 (mean ± SD) aneuploid CECs/HD (Fig. 3g; min 0/max 13 cells, n = 47), whereas cancer patients (n = 133) had an average number of 8.8 ± 14.3 (min 1/max 144 cells) aneuploid CECs/patient. The difference in cell number/subject (patient *vs* HD) was statistically significant (*p* < 0.001). Shown in Fig. 3h, further analysis of CECs in two different population of patients (107 of CD31^+^/Vim^−^ *vs* 26 of CD31^+^/Vim^+^) indicated that, the CD31^+^/Vim^−^ population of 107 patients had an average cell number of 9.1 ± 15.3 CECs/subject. Analysis of those two different phenotypes of CECs (CD31^+^/Vim^−^ *vs* CD31^+^/Vim^+^) in the same population of 26 patients revealed the average cell count of 5.4 ± 8.9 of CD31^+^/Vim^−^ cells/patient (blue), and 2.3 ± 1.8 CD31^+^/Vim^+^ CECs/patient (red), respectively. The quantity difference in CD31^+^/Vim^−^ phenotype CECs per subject between two different populations of patients (107 *vs* 26) was statistically significant (*p* < 0.01). However, no significant statistical difference (*p* > 0.05) in cell number per subject between two diverse phenotype of CECs (CD31^+^/Vim^−^ *vs* CD31^+^/Vim^+^) was observed in this preliminary study performed on the same population of small cohorts of 26 patients.

Additional analysis indicated that 2 of cancer patients were found to have a total of 4 aneuploid cells and 6 CTMs displaying a unique triple positive phenotype of CD31^+^/Vim^+^/EpCAM^+^.

In view of inter- and intra-patient heterogeneity of aneuploid CECs in cancer patients and healthy blood donors, substantial characterization of the precise subtype compositions of aneuploid CD31^+^ cells in both, normal and malignant conditions, remains to be further thoroughly conducted on a large cohort of healthy blood donors and cancer patients, which will help address biologic and pathologic significance of aneuploid CD31^+^ CECs in carcinoma patients and healthy subjects, respectively.

## Conclusions

The novel SE-iFISH provides a unique strategy for expeditious and effective enrichment, *in situ* phenotypic as well as karyotypic co-detection of various non-hematopoietic CRCs in cancer patients or metastatic mouse model of patient derived xenograft (mPDX)^33^, which allows for classification of CTCs and CECs into diverse subtypes upon biomarker expression as well as chromosome ploidy, and each subtype of CRCs may correlate with distinct clinical outcome, such as tumor angiogenesis, cancer metastasis, relapse and therapeutic drug resistance^7, 33^.

It remains so far unclear how aneuploid CTCs and CECs correlate with tumor progression and metastases formation in patients with malignancies. In addition to existence of individual aneuploid CTCs and tumor CECs identified in carcinoma patients in this study, surprisingly, some aneuploid neoplastic cells and CTMs in cancer patients were found to have a non-reported, unique phenotype of CD31^+^/EpCAM^+^/Vimentin^+^. Given the reported significant relevance of tumor endothelial cells related angiogenesis to tumor metastasis^1, 9, 39, 40^, as well as high metastatic potential of CTMs in carcinoma patients^2, 44^, extensive co-investigation of aneuploid CTCs and CD31^+^ tumor CECs, will help further illustrate how those diverse subtypes of aneuploid malignant cells have a cross-talk and functional interplay with tumor angiogenesis, progression and metastasis, respectively.

## Methods

### Patients

The studies, including all methods, were carried out in accordance with the Declaration of Helsinki Principles. All the experimental protocols, including patient recruitment, blood collection and subsequent SE-iFISH processing were approved by the Ethics Review Committees (ERC) of Shanghai General Hospital (SGH), Shanghai, China. Consent forms, including indicated experimental protocols, signed by all enrolled patients were approved by the ERC of SGH. The written informed consent forms were received from all subjects prior to enrollment in the study.

### Subtraction Enrichment (SE)

Significant improvement was made in this study on the prototype of SE protocol previously published^14^. Experiments were performed according to the manufacture’s updated instruction (Cytelligen, San Diego, CA, USA). Briefly, 6 ml of blood were collected into a tube containing ACD anti-coagulant (Becton Dickinson, Franklin Lakes, NJ, USA). Samples were stored at room temperature for no more than 48 hrs prior to processing. Blood samples were centrifuged at 200 × g for 15 min at room temperature. Supernatant plasma was kept at −80 °C for future nucleic acid and plasma protein analyses. Sedimented blood cells were gently mixed with 3.5 ml of hCTC buffer, followed by loading on the non-hematopoietic cell separation matrix in a 50 ml tube, and subsequent centrifugation at 450 × g for 5 min. Solution containing WBCs and tumor cells but lacking RBCs was collected into a 50 ml tube, and then incubated with 300 μl of immuno-magnetic beads conjugated to a cocktail of anti-leukocyte mAbs, at room temperature for 30 min with gentle shaking. WBCs bound to immuno-beads were depleted using a magnetic separator. Solutions free of magnetic beads were collected into a 15 ml tube, followed by adding hCTC buffer to 14 ml. Samples were spun at 500 × g for 4 min at room temperature. Supernatants were aspirated down to 50 μl. Sedimented cells in 50 μl solution were gently resuspended, followed by subjection to primary tumor cell culture, viability examination, immunofluorescence staining, or drying monolayer of cells mixed with the special fixative on the Cytelligen coated and formatted CTC slides for subsequent iFISH analyses.

100 to 150 of the indicated tumor cells were spiked into 6 ml of blood each time for subsequent iFISH validation study, and 80 to 100 SK-BR-3 breast cancer cells were spiked into the same volume of blood each time for viability examination described below.

### iFISH

Six color iFISH developed in this study was performed according to the manufacture’s updated protocol (Cytelligen). Briefly, dried monolayer cells on the coated and formatted CTC slides (Cytelligen) were rinsed and incubated with PBS at room temperature for 3 min, followed by hybridization with Vysis chromosome 8 centromere probe (CEP8) SpectrumOrange (Abbott Laboratories, Abbott Park, IL, USA) for 4 hrs using a S500 StatSpin ThermoBrite Slide Hybridization/Denaturation System (Abbott Molecular, Des Plaines, IL, USA). Samples were subsequently incubated with Alexa Fluor (AF) 488 (green), 594, (red), 647 (equal to Cy5) and 750 (equal to Cy7) respectively conjugated to the mAbs recognizing indicated targets, including CD45, CD31, EpCAM, CK18, HER2, Vimentin, or PD-L1, etc., at room temperature for 20 min in dark^27, 36^. After washing, samples were mounted with mounting media containing DAPI (Vector Laboratories, Burlington, CA, USA), and subjected to automated CTC image scanning and analyses. Upon heterogeneous subcellular localization of a variety of tumor biomarkers, it might be necessary to empirically optimize and perform immunofluorescence staining of the specific targeted protein either before or after FISH.

### Automated CRC scanning and image analysis performed by Metafer-i•FISH^®^

iFISH CTCs/CECs on slides were scanned using an automated Metafer-i•FISH^®^ CRC scanning and image analyzing system newly co-developed by Carl Zeiss, MetaSystems, and Cytelligen. The coated and formatted iFISH CRC slides (Cytelligen) were automatically loaded on a Zeiss fluorescence microscope (AXIO Imager. Z2), and subsequently subjected to automated X-Y scanning with cross Z-sectioning of all cells performed at 1 μm steps of depth. Scanning was performed in 6 fluorescence color channels. Positive target cells are defined as DAPI^+^, CD45^−^, tumor biomarker(s)^+^ and/or CD31^+^ with diploid or aneuploid chromosome 8.

Following high through-put CRC scanning, image acquiring and processing, subsequent automated CRC classification and statistical analyses are performed upon cell size, cell cluster, quantified immunostaining intensity of multiple tumor biomarkers expression, and chromosome ploidy, etc.

### Viability examination of the tumor cells enriched from blood

As a proof-of-concept, 80 to 100 of SK-BR-3 breast cancer cells were spiked into 6 ml of blood, followed by subtraction enrichment. Enriched tumor cells were incubated with AF488 labeled anti-HER2 mAb for 20 min at room temperature in dark. After incubation, cells were centrifuged at 950 × g for 4 min. Sedimented cells were resuspended with 100 μl PBS, and incubated with 10 μl of 7-aminoactionomycin D (7-AAD, 5 μg/ml) (Life Technologies, Carlsbad, CA, USA), at room temperature for 10 min, then with 5 μl of nucleus staining dye Hoechst 33342 (0.5 mg/ml, Thermo Fisher Scientific, Skokie, IL, USA) for additional 15 min at room temperature in dark. Cells were washed twice with PBS, followed by microscopic analysis. Nuclei of necrotic cells were stained red with 7-AAD.

### Statistical analysis

All statistical analyses, including chi-square test, Mann-Whitney U test, were performed with SPSS 18.0. *p* < 0.05, *p* < 0.01 and *p* < 0.001 are statistically significant, very significant, and extremely significant, respectively. All *p* values were two-tailed.

### Data Availability

The datasets generated during and/or analysed during the current study are available from the primary corresponding author (PL) on reasonable request.
